# Partial Purification and Characterization of a Heat Stable *α*-Amylase from a Thermophilic Actinobacteria, *Streptomyces* sp. MSC702

**DOI:** 10.1155/2014/106363

**Published:** 2014-10-08

**Authors:** Renu Singh, Vijay Kumar, Vishal Kapoor

**Affiliations:** ^1^Laboratory of Microbiology, Department of Botany, Christ Church College, Kanpur 208001, India; ^2^Department of Civil Engineering, Indian Institute of Technology, Kanpur 208016, India

## Abstract

A partial purification and biochemical characterization of the *α*-amylase from *Streptomyces* sp. MSC702 were carried out in this study. The optimum operational conditions for enzyme substrate reaction for amylolytic enzyme activity from the strain were evaluated. The optimum pH, temperature, and incubation period for assaying the enzyme were observed to be 5.0, 55°C, and 30 min, respectively. The extracellular extract was concentrated using ammonium sulfate precipitation. It was stable in the presence of metal ions (5 mM) such as K^+^, Co^2+^, and Mo^2+^, whereas Pb^2+^, Mn^2+^, Mg^2+^, Cu^2+^, Zn^2+^, Ba^2+^, Ca^2+^, Hg^2+^, Sn^2+^, Cr^3+^, Al^3+^, Ag^+^, and Fe^2+^ were found to have inhibitory effects. The enzyme activity was also unstable in the presence of 1% Triton X-100, 1% Tween 80, 5 mM sodium lauryl sulphate, 1% glycerol, 5 mM EDTA, and 5 mM denaturant urea. At temperature 60°C and pH 5.0, the enzyme stability was maximum. *α*-amylase retained 100% and 34.18% stability for 1 h and 4 h, respectively, at 60°C (pH 7.0). The enzyme exhibited a half-life of 195 min at 60°C temperature. The analysis of kinetic showed that the enzyme has *K*_*m*_ of 2.4 mg/mL and *V*_max_ of 21853.0 *μ*mol/min/mg for soluble potato starch. The results indicate that the enzyme reflects their potentiality towards industrial utilization.

## 1. Introduction

Microbial amylases are among the most important hydrolytic enzymes and have been studied extensively. This group of enzymes represents one of the three largest groups of industrial enzymes and accounts for approximately 25–33% of the world enzyme market, in second place after proteases [1]. They have numerous applications in the industrial processing of different items, namely, starch liquefaction process [2], improve flour in the baking industry, produce modified starch for paper industry [3] and as an ingredient in automatic dishwasher and laundry detergent formulations [4].

Various microorganisms in nature, mostly fungi and bacteria, have complex amylolytic enzyme systems that are associated with starch decomposition and are responsible for hydrolyzing starch into simple sugars. Recently, several members of group actinobacteria provided a remarkable alternative to these traditional groups [5–7]. Application of thermophilic microorganisms to produce enzyme for industrial use is a general practice because they provide broader temperature range and higher thermostability compared to enzymes from mesophilic microorganisms. The utilization of thermophilic actinobacteria in the cellulolytic, laccase, and xylanase enzyme production was well categorized [8–10]. Moreover, no report was published for the characterization of thermostable *α*-amylase isolated by thermophilic actinobacteria.

The previous publications by us covered the screening of strain* Streptomyces* sp. MSC702 and the optimization of the fermentation medium [11, 12] for the production of *α*-amylase enzyme. *α*-Amylase production by* Streptomyces* sp. MSC702 is significant as it is a thermostable and Ca^2+^-ion independent and exhibits a high degree of raw starch digestibility [12]. The partial purification and characterization of the enzyme as well as some kinetic data from* Streptomyces* sp. MSC702 are presently reported.

## 2. Materials and Methods

### 2.1. Actinobacteria and Culture Conditions

The amylolytic* Streptomyces* sp. MSC702 isolated from the mushroom compost in India was used as biological material [11]. Strain MSC702 was isolated on M medium agar [13] for 45°C at pH 7.0. M medium was modified with 1% (v/v) trace metal salt solution [14]. The strain was maintained on modified M medium agar slants at 4°C. All the culture media were autoclaved at 121°C (15 lbs) for 20 min.

### 2.2. Improvement of *α*-Amylase Production

*α*-Amylase production in submerged fermentation (SmF) was carried out in 250 mL Erlenmeyer flask using basal medium containing 1.0% rice bran, 2.0% wheat bran, 0.1% K_2_HPO_4_, 0.1% (NH_4_)_2_SO_4_, 0.1% NaCl, and 0.1% MgSO_4_ *·*7H_2_O at pH 7.0. Cotton plugged flask was autoclaved at 121°C for 20 min and cooled. The medium was inoculated with 1% inoculum and incubated at 50°C for 48 h. Samples were harvested by filtering through Whatman filter papers 1 (qualitative circles, 125 mm diameter) and centrifuged at 5,000 g for 20 min at 4°C; the cell-free supernatant (crude enzyme) was used for *α*-amylase assay.

### 2.3. Amylase Assay and Protein Determination

*α*-Amylase activity was estimated by analyses of reducing sugar released during hydrolysis of 1.0% (w/v) starch in 0.1 M phosphate buffer (pH 7.0) by enzyme (cell-free supernatant) incubated at 50°C for 10 min. The amount of reducing sugar level released in the mixture was determined by the dinitrosalicylic acid (DNS) method [15]. Absorbance at 550 nm was recorded by using UV-visible spectrophotometer (UV-1700 Pharmaspec Shimadzu) and activity was calculated from a standard curve using maltose as the standard. One unit (U) of enzyme activity was defined as the amount of enzyme required for the liberation of 1 *μ*mol reducing sugar as maltose per minute under standard assay conditions. Total protein was estimated using BSA (bovine serum albumin) as standard, as described by Lowry et al. [16]. All experiments were carried out in triplicate and the data presented are average values.

### 2.4. Amylase Purification

The various steps of enzyme purification were carried out at 4°C unless otherwise mentioned. The crude enzyme was treated with solid ammonium sulphate with continuous overnight stirring and separation into the following saturation ranges: 0–20%, 20–40%, 40–60%, and 60–80%. The precipitates collected by centrifugation (10,000 g for 15 min) were dissolved in 0.1 M phosphate buffer, pH 7.0. The enzyme solution was dialysed against the same buffer for 12 h with several changes to remove the salt and assayed by the method described by Roe [17].

### 2.5. Estimation of Optimum Operational Conditions for Amylolytic Enzyme Activity

The optimum incubation temperature was examined by carrying the enzyme-substrate reaction for 10 min at different temperatures (50–90°C) keeping constant pH 7.0 (0.1 M phosphate buffer). Further optimum reaction time was determined by carrying the enzyme-substrate reaction at optimum temperature (55°C) and constant pH 7.0 (0.1 M phosphate buffer). Enzyme activity was checked for 65 min at 5 min interval and was expressed as percentage relative activity.

The pH optima of the *α*-amylase were estimated by preparing the reaction mixture with various pH buffers and assayed for 10 min at 55°C. Three buffers (0.1 M) were used for different pH, that is, phosphate-citrate buffer for pH 3.0, 4.0 and 5.0, phosphate buffer for pH 6.0, 7.0 and 8.0, and glycine-NaOH buffer for pH 9.0, 9.8 and 10.6. Enzyme activity was expressed as percentage relative activity.

### 2.6. Characterization of *α*-Amylase

#### 2.6.1. Effect of Temperature and pH on Enzyme Stability

To estimate thermostability, crude enzyme was preincubated for 30 min, at different temperatures (50–85°C) before enzyme assay, and promptly cooled on ice and residual activity was determined under standard assay conditions. The half-life of *α*-amylase was determined by incubating the crude enzyme at 60°C and residual activity was measured after every 15 min for 240 min (4 h) under standard assay conditions.

Effect of various pH buffers (3–10.6) on enzyme stability was studied by incubating the enzyme with various pH buffers, as stated above, for 30 min at 60°C before enzyme assay and the residual activity was determined under standard assay conditions. Effect of pH on enzyme thermostability was also determined at 60°C by measuring the residual activity after every 15 min for 240 min (4 h) under standard assay conditions.

#### 2.6.2. Effect of Various Reagents on Enzyme Activity

Effect of various additives such as salts of 16 metal ions (5 mM) (K^+^, Ag^+^, Pb^2+^, Mn^2+^, Mg^2+^, Fe^2+^, Co^2+^, Cu^2+^, Zn^2+^, Ba^2+^, Mo^2+^, Ca^2+^, Hg^2+^, Sn^2+^, Cr^3+^, and Al^3+^), 4 surfactants {Triton X-100 (1%), Tween 80 (1%), sodium lauryl sulphate (5 mM), and glycerol (1%)}, chelating agent EDTA (5 mM), and denaturant urea (5 mM) on enzyme activity was tested by incorporating 1 mL solution of each additive in enzyme-substrate reaction mixture. The reaction was carried out for 30 min. Enzyme activity was measured under standard assay conditions. Enzyme activity was determined as percentage relative activity of control (without additives) considered as having 100%.

#### 2.6.3. Steady State Kinetics Measurement

Kinetic parameters for *α*-amylase were determined by incubating the crude enzyme with various concentrations (0.5–8.0 mg/mL) of soluble potato starch under standard assay conditions. The Michaelis-Menten constant (*K*_*m*_) and maximum velocity (*V*_max⁡_) values were determined from Lineweaver-Burk plots. The *K*_*m*_ and *V*_max⁡_ values were calculated from the kinetic data using the “GraphPad Prism” software.

## 3. Results and Discussion 

### 3.1. Optimum Operational Conditions

**Figure 1:**
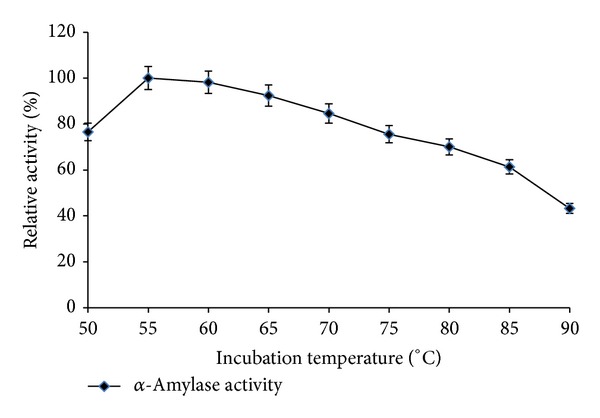
Effect of different incubation temperatures on enzyme activity (10 min incubation).

**Figure 2:**
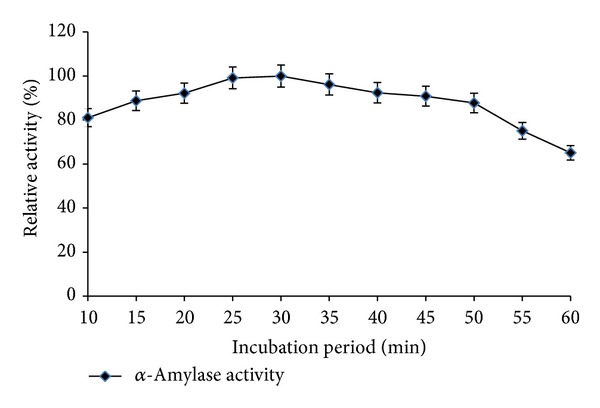
Effect of different incubation periods on enzyme activity (at 55°C for *α*-amylase).

The optimum temperature for the *α*-amylase activity from* Streptomyces* sp. MSC702 was in a wide range of 50–75°C (retained >74% relative activity at the temperature upto 75°C) with maximum activity at 55°C (Figure 1). However, at temperatures 85°C and 90°C, the retained relative activity of *α*-amylase was 61.33% and 43.26%, respectively. Enzyme-substrate reaction was maximally active in the range of 10 min to 50 min (>80% relative activity) with maximum *α*-amylase activity achieved in 30 min at 55°C (Figure 2). There was a remarkable decrease in *α*-amylase activity after 50 min incubation. The increase in incubation period might induce conformational changes in 3D structure of the enzyme affecting its substrate affinity. Chakraborty et al. [18] reported a drastic decrease in *α*-amylase activity at 90°C with maximum activity at 50°C from* Streptomyces *sp. D1. Syed et al. [19] reported optimal activity at 45°C for *α*-amylase from* S. gulbargensis*. Results from present study provide lines of evidence that *α*-amylase from* Streptomyces* sp. MSC702 could be a good candidate for the efficient liquefaction of gelatinized starch.

**Figure 3:**
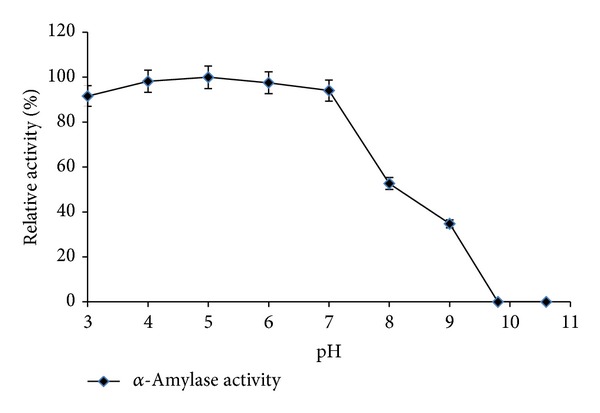
Effect of different pH on enzyme activity with 10 min incubation (at 55°C for *α*-amylase).

The optimum pH for *α*-amylase activity from* Streptomyces* sp. MSC702 ranged from pH 3.0 to 7.0 (retained >91% activity) with a maximum activity at pH 5.0 (Figure 3). Although a decline in enzyme activity was observed between pH 8.0 and pH 9.0, the enzyme was still active at pH 8.0 and 9.0, retaining its 52.71 and 34.78% activity. A complete loss in the enzyme activity was observed above pH 9.8. Activity of *α*-amylase at low pH range is very important for industrial applications [20]. The application of liquefying amylases that are active and stable around the saccharification pH is attractive to avoid or reduce the use of acid to lower the pH from liquefying to saccharifying range and also to simplify the procedures during downstream processing. Further, the use of *α*-amylases that operate at lower pH values reduces the formation of some by-products, such as maltulose, which is usually produced at higher operation pH [21]. Ammar et al. [22] reported optimum pH 6.0-7.0 for* Streptomyces* sp. *α*-amylase. In contrast, Chakraborty et al. [18] and Syed et al. [19] reported optimum activity at pH 9.0 for* Streptomyces* sp. D1 and* S. gulbargensisα*-amylases, respectively.

### 3.2. Effect of Metal Ions and Surfactants on *α*-Amylase Activity

The variety of ways by which metal ions affect enzyme catalysis that is, by modifying the electron flow in the enzyme substrate reaction or by changing the orientation of the substrate with reference to the functional group at active site. Metal ions accept or donate electrons and act as electrophiles, mask nucleophiles to prevent unwanted side reactions, bind enzyme and substrate by coordinate bonds, hold the reacting groups in the required 3D orientation, and simply stabilize a catalytically active conformation of the enzyme [23]. Effect of metal ions and other additives on the activity of *α*-amylase by* Streptomyces* sp. MSC702 and its comparison with the earlier reports are presented in Table 1.

Among the various metal salts and chemical reagents tested, it was found that the *α*-amylase activity was almost completely inhibited by (5 mM) Pb^2+^, Mn^2+^, Mg^2+^, Cu^2+^, Zn^2+^, Ba^2+^, Ca^2+^, Hg^2+^, Sn^2+^, Cr^3+^, and Al^3+^ metal ions. Ag^+^ and Fe^2+^ inhibited *α*-amylase activity up to 40.27% and 50.96%, respectively. Metal ions such as K^+^ (154.32% relative activity), Co^2+^ (391.82% relative activity), and Mo^2+^ (154.81% relative activity) strongly stimulated *α*-amylase activity. The effect of Co^2+^ ions on *α*-amylase activity varies drastically with strain to strain of* Streptomyces*. Chakraborty et al. [18] reported stimulation while Syed et al. [19] reported inhibition of *α*-amylase activity in* Streptomyces* sp. D1 and* S. gulbargensis*, respectively, in the presence of Co^2+^ ions. The unusual behavior of the enzymes for Co^2+^ ions might be related to its special structure and the mechanism of action behind this is subject to further research. Metal ions such as K^+^ strongly stimulated the *α*-amylase activity, similar to the report of Zhang and Zeng [24].

Most of the amylases reported till date are metal ion dependent enzymes and Ca^2+^ ions known to be a stabilizer for amylases isolated by various microorganisms. In case of the strain MSC702, the role of Ca^2+^ ions is inhibitory, showing its applicability in the confectionary industry, particularly in the making of fructose syrups. When the strain MSC702 *α*-amylase was incubated with Pb^2+^, Mg^2+^, Cu^2+^, Zn^2+^, Ba^2+^, Ca^2+^, Hg^2+^, and Fe^2+^, the activity reduced drastically, similar to the report of Uyar et al. [25]. The inhibition of Hg^2+^ indicates the presence of indole amino acid residues in enzyme [26]. In the previous reports of Chakraborty et al. [18] and Syed et al. [19], *α*-amylases from* Streptomyces* sp. D1 and* Streptomyces gulbargensis* were also strongly inhibited by Hg^2+^ ion. The inhibition due to Cu^2+^ ions reflected the competition between the exogenous and protein associated cations. Inhibition with Zn^2+^ determines the thermostable nature of enzyme [27].

With respect to the effect of surfactants, *α*-amylase showed 40.92%, 47.37%, 34.21%, and 36.52% relative activity in presence of Triton X-100 (1% v/v), Tween 80 (1% v/v), SLS (5 mM), and glycerol (1% v/v), respectively. In order to have applications in detergent industries, amylase must be stable to various detergent ingredients, such as surfactants. The obtained results show a reasonably good stability in the enzyme activity using different detergents at 1% v/v, suggesting that the enzyme has a potential in starch liquefaction and detergent industry. Chelating agent EDTA completely inhibited *α*-amylase activity, similar to the previous report of Chakraborty et al. [18]. The inhibitory effect of EDTA provides evidence that the enzyme's activity is metal dependent. The denaturation of the original *α*-amylase activity with urea (23.93% relative activity) in the present study concluded that the enzyme consists of hydrophobic amino acid composition [28].

### 3.3. Thermal and pH Stability

**Figure 4:**
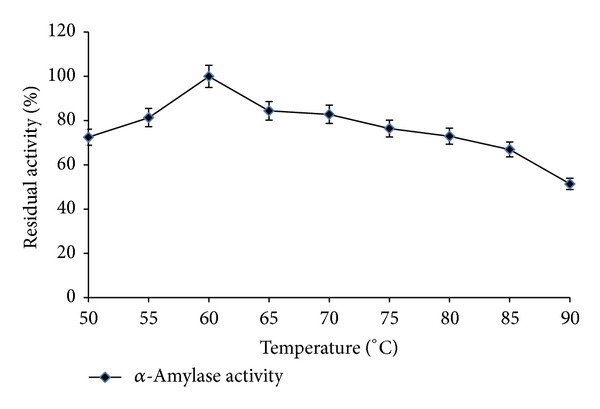
Effect of different temperatures on enzyme stability (preincubation for 30 min, pH 7.0).

**Figure 5:**
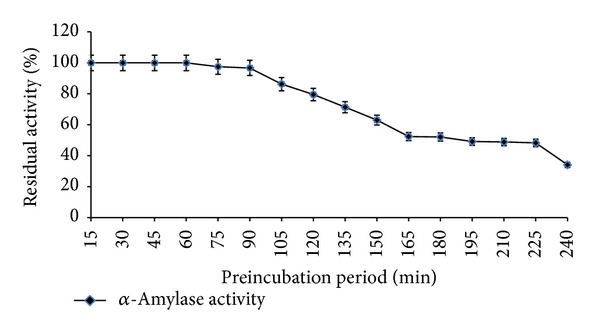
Effect of optimum temperature (60°C) on enzyme stability with varying preincubation period (pH 7.0).

Thermal stability is a very important aspect of industrial enzymatic bioreactors. Profiles of thermal stability of* Streptomyces* sp. MSC702 *α*-amylase between 50 and 90°C are presented in Figures 4 and 5. Temperature stability profile showed that *α*-amylase had maximum stability at 60°C and retained 51.38% activity at 90°C (Figure 4). *α*-Amylase was 100% stable for 1 h at 60°C and retained 34.18% activity after 4 h of preincubation (Figure 5). Half-life (*t*_1/2_) of *α*-amylase was recorded 195 min at 60°C. From these results, the enzyme seemed to have considerable thermostability, which can be favorable in industrial operations for traditional brewing and food processing [29]. In contrast, Syed et al. [19] and Ammar et al. [22] reported instability at higher temperature (>60°C) for* Streptomyces* spp. *α*-amylases.

**Figure 6:**
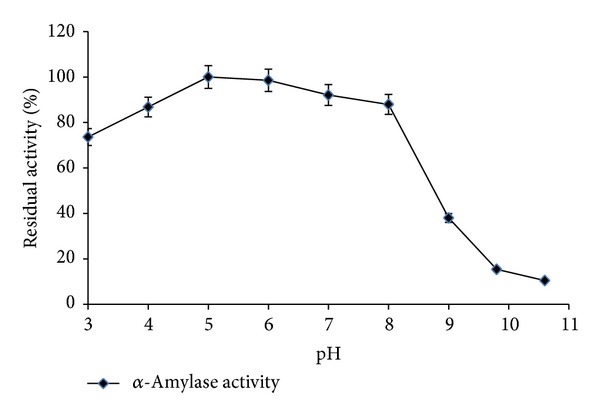
Effect of different pHs on enzyme stability at 60°C (preincubation for 30 min).

**Figure 7:**
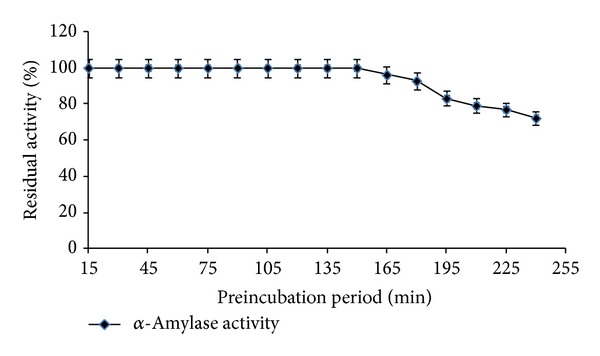
Effect of pH (pH 5.0 for *α*-amylase) on enzyme stability at 60°C with varying preincubation period.

Profiles of pH stability of* Streptomyces* sp. MSC702 *α*-amylase at 60°C are presented in Figures 6 and 7. *α*-Amylase showed broad pH stability (pH 3.0–8.0) profile with maximum stability at pH 5.0 (Figure 6). The pH stability profile showed that enzyme was highly stable at acidic pH range. However, the enzyme was 87.93% and 37.98% stable at pH 8.0 and 9.0, respectively; after this, a remarkable loss in stability occurred. *α*-Amylase was completely stable at 60°C with pH 5.0 after 150 min preincubation. However, *α*-amylase retained 72.19% stability after 4 h preincubation (Figure 7). Similar to the present study, Ammar et al. [22] observed the maximum enzyme stability at pH 5.0–7.0 for* Streptomyces* sp. *α*-amylase. In contrast, Chakraborty et al. [18] and Syed et al. [19] reported stability of enzyme at alkaline pH range (7.0–11.0) for* Streptomyces *sp. D1 and* S. gulbargensisα*-amylases, respectively. Chakraborty et al. [18] found stability of *α*-amylase for 6–48 h at pH 7.0–11.0.

### 3.4. Partial Purification of *α*-Amylase by Ammonium Sulphate Precipitation

Several industrial processes are carried out using whole cells as the source of enzymes but the efficiency can be improved using isolated and purified enzymes. However, the criteria for selection of a particular method of isolation and purification depend on its end use. Various steps of ammonium sulphate precipitation followed by dialysis for partial purification of *α*-amylase from* Streptomyces *sp. MSC702 are summarized in Table 2.

In the present study, the ammonium sulphate precipitation (40–60% saturation) followed by dialysis of crude *α*-amylase yielded 56.58% of the enzyme with 2.98-fold purification. Yang and Liu [30] recovered 55% *α*-amylase of* Thermobifida fusca* NTU22 with 1.3-fold purification by ammonium sulphate precipitation. Mollania et al. [31] purified 1.3-fold *α*-amylase of* Geobacillus *LH8 strain with 4.29% yield by ammonium sulphate precipitation. Kikani and Singh [32] yielded 34.29% *α*-amylase of* Bacillus amyloliquifaciens* TSWK1-1 with 4.29-fold purification. In the present work, a good yield and purification of *α*-amylase was achieved compared to previous reports.

### 3.5. Kinetic Determinations

**Figure 8:**
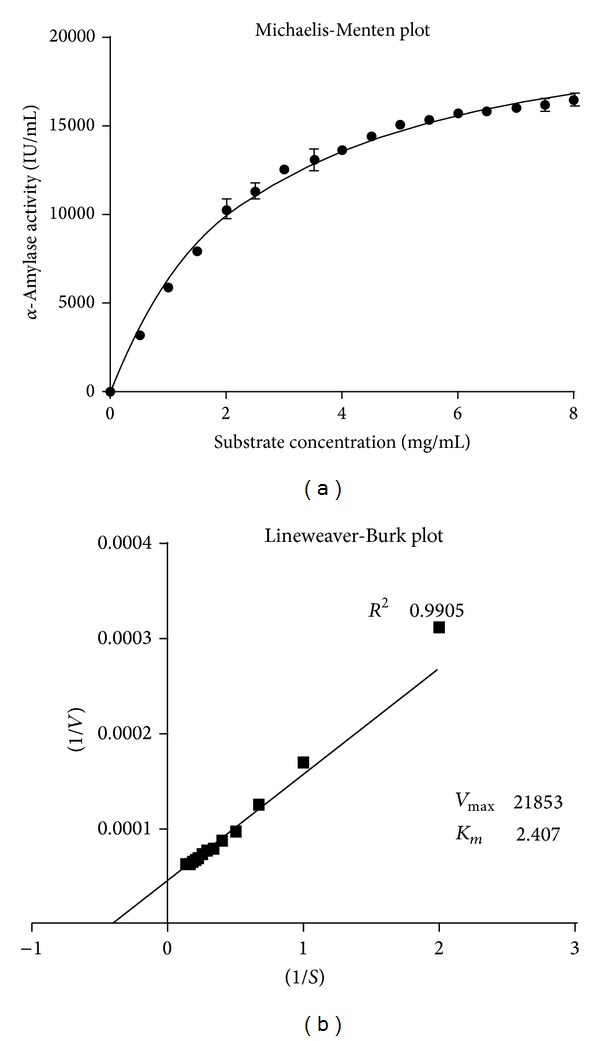
(a) Michalis-Menten plot and (b) Lineweaver-Burk plot for *K*_*m*_ and *V*_max⁡_ values of the *α*-amylase in the presence of different concentrations of soluble starch.

From an industrial viewpoint, it is desirable to choose an enzyme which will have the fastest reaction rate per unit amount of enzyme as this indicates the maximum effect for the minimum amount of added catalyst. The Michaelis-Menten plot and Lineweaver-Burk plot for *α*-amylase by* Streptomyces* sp. MSC702 are depicted in Figures 8(a) and 8(b).

Present results showed that the Michaelis-Menten constant (*K*_*m*_) and *V*_max⁡_ value for *α*-amylase were 2.407 mg/mL and 21853.0 *μ*mol/min/mg, respectively, (Figures 8(a) and 8(b)). It is difficult to compare the kinetic values of *α*-amylase obtained by other researchers in view of the usage of different starch concentrations or different assay conditions. Shafiei et al. [33] found *K*_*m*_ and *V*_max⁡_ values for *α*-amylase by* Nesterenkonia *sp. strain *F* to be 4.5 mg/mL and 1.18 mg/mL/min, respectively. Kikani and Singh [32] reported *K*_*m*_ and *V*_max⁡_ values for *α*-amylase by* Bacillus amyloliquifaciens* TSWK1-1 as 0.6 mg/mL and 2632 mol/mL/min, respectively.

## 4. Conclusions

For a successful biotechnological application, purification and characterization are important prerequisites. *α*-Amylase enzyme related to starch degradation was purified and characterized from a novel thermophilic actinobacteria strain,* Streptomyces* sp. MSC702. The strain had a broad range of temperature stability showing its suitability in the industrial applications where temperature plays a critical role. A plausible assay mechanism of starch degradation by the enzymes was also suggested.

## Figures and Tables

**Table 1:** Comparative analysis of the effect of different additives on enzyme stability.

Additives	*α*-Amylase relative activity (%)					
Metal ions	A_1_^#^	A_2_^#^	A_3_^#^	A_4_^#^	A_5_^#^	A_*x*_^#^
KCl (5 mM)	—	100	—	108	—	154.32
AgCl (5 mM)	—	—	—	—	—	69.23
Pb(NO_3_)_2_ (5 mM)	—	—	40	—	—	8.65
MnSO_4_ *·*H_2_O (5 mM)	84.37	110	68	143	—	0
MgSO_4_ *·*7H_2_O (5 mM)	87.38	109.87	46	113	—	7.21
FeSO_4_ *·*7H_2_O (5 mM)	—	56.67	43	107	0	49.04
CoCl_2_ (5 mM)	44.78	104.50	—	138	76.46	391.82
CuSO_4_ (5 mM)	34.61	108.79	50	142	38.95	0
ZnSO_4_ (5 mM)	84.78	—	45	106	0	0
BaCl_2_ (5 mM)	—	—	—	—	58.82	0
(NH_4_)_6_Mo_7_O_24_ (5 mM)	—	—	—	—	—	154.81
CaCl_2_ (5 mM)	100	125	87.50	115	185	14.90
HgCl_2_ (5 mM)	31.45	64.23	29	72	36.46	0
SnCl_2_ (5 mM)	—	—	—	—	—	0
CrO_3_ (5 mM)	—	—	—	—	—	0
AlCl_3_ (5 mM)	—	—	—	—	—	0

Surfactants						
Triton X-100 (1%)	—	—	—	—	—	40.92
Tween 80 (1%)	—	—	—	—	—	47.37
SLS (5 mM)	—	—	—	—	—	34.21
Glycerol (1%)	—	—	—	—	—	36.52

Chelating agent						
EDTA (5 mM)	95.23	2.13	—	85	—	0

Denaturant						
Urea (5 mM)	16.45	9.45	—	—	—	23.93

∗Control	100	100	100	100	100	100
References	[26]	[34]	[25]	[24]	[35]	Current study

*Enzyme without any additive; A_1_^#^ = *Streptomyces* strain A3; A_2_^#^ = *Saccharopolyspora* sp. A9; A_3_^#^ = *Bacillus subtilis*; A_4_^#^ = *Nocardiopsis* sp. 7326; A_5_^#^ = *Bacillus cereus* GA6; A_*x*_^#^ = *Streptomyces* sp. MSC702 (enzyme preincubated at 60°C with 30 min); —: Not tested.

**Table 2:** Partial purification of *α*-amylase by ammonium sulphate precipitation followed by dialysis.

Fraction	Total protein (mg)	Total activity (IU)	Specific activity (IU/mg)	Purification (Fold)	Yield (%)
Raw extract	4821.3	450000	93.33	1	100
*F* (0–20%)	801.5	64285.7	80.2	0.86	14.29
*F* (20–40%)	561.05	46250.0	82.43	0.88	10.27
*F* (40–60%)	914.96	254651.2	278.32	2.98	56.58
*F* (60–80%)	136.63	12500.9	91.49	0.98	2.7

## References

[1] Nguyen QD, Rezessy-Szabó JM, Claeyssens M, Stals I, Hoschke Á (2002). Purification and characterisation of amylolytic enzymes from thermophilic fungus *Thermomyces lanuginosus* strain ATCC 34626. *Enzyme and Microbial Technology*.

[2] Gupta R, Gigras P, Mohapatra H, Goswami VK, Chauhan B (2003). Microbial *α*-amylases: a biotechnological perspective. *Process Biochemistry*.

[3] Pandey A, Nigam P, Soccol CR, Soccol VT, Singh D, Mohan R (2000). Advances in microbial amylases. *Biotechnology and Applied Biochemistry*.

[4] Kim TU, Gu BG, Jeong JY, Byun SM, Shin YC (1995). Purification and characterization of a maltotetraose-forming alkaline (alpha)-amylase from an alkalophilic Bacillus strain, GM8901. *Applied and Environmental Microbiology*.

[5] Igbokwe GE, Ngobidi KC, Iwuchukwu NP (2013). Production of alpha-amylase from mixed *Actinomyces* spp. cultured at room temperature using Nelson’s colorimetric method. *Asian Journal of Biological Sciences*.

[6] Salahuddin K, Prasad R, Kumar S, Visavadia MD (2011). Isolation of soil thermophilic strains of actinomycetes for the production of *α*-amylase. *African Journal of Biotechnology*.

[7] Singh R, Kapoor V, Kumar V (2012). Utilization of agro-industrial wastes for the simultaneous production of amylase and xylanase by thermophilic actinomycetes. *Brazilian Journal of Microbiology*.

[8] Saratale GD, Saratale RG, Oh SE (2012). Production and characterization of multiple cellulolytic enzymes by isolated *Streptomyces* sp. MDS. *Biomass & Bioenergy*.

[9] Techapun C, Sinsuwongwat S, Poosaran N, Watanabe M, Sasaki K (2001). Production of a cellulase-free xylanase from agricultural waste materials by a thermotolerant *Streptomyces* sp. *Biotechnology Letters*.

[10] Ben Younes S, Sayadi S (2011). Purification and characterization of a novel trimeric and thermotolerant laccase produced from the ascomycete *Scytalidium thermophilum* strain. *Journal of Molecular Catalysis B: Enzymatic*.

[11] Singh R, Kapoor V, Kumar V (2011). Influence of carbon and nitrogen sources on the *α*-amylase production by a newly isolated thermotolerant *Streptomyces* sp. MSC702 (MTCC 10772). *Asian Journal of Biotechnology*.

[12] Singh R, Kapoor V, Kumar V (2012). Production of thermostable, Ca^+2^-independent, maltose producing *α*-amylase by *Streptomyces* sp. MSC702 (MTCC 10772) in submerged fermentation using agro-residues as sole carbon source. *Annals of Microbiology*.

[13] Obi SKC, Odibo FJC (1984). Partial purification and charaterization of a thermostable actinomycete *β*-amylase. *Applied and Environmental Microbiology*.

[14] Techapun C, Charoenrat T, Poosaran N, Watanabe M, Sasak K (2002). Thermostable and alkaline-tolerant cellulase-free xylanase produced by thermotolerant *Streptomyces* sp. Ab106. *Journal of Bioscience and Bioengineering*.

[15] Miller GL (1959). Use of dinitrosalicylic acid reagent for determination of reducing sugar. *Analytical Chemistry*.

[16] Lowry OH, Rosenbrough NJ, Farr AL, Randall RJ (1951). Protein measurement with the Folin phenol reagent. *The Journal of Biological Chemistry*.

[17] Roe S (2001). *Purification and Concentration by Precipitation*.

[18] Chakraborty S, Khopade A, Kokare C, Mahadik K, Chopade B (2009). Isolation and characterization of novel *α*-amylase from marine *Streptomyces* sp. D1. *Journal of Molecular Catalysis B: Enzymatic*.

[19] Syed DG, Agasar D, Pandey A (2009). Production and partial purification of *α*-amylase from a novel isolate *Streptomyces* gulbargensis. *Journal of Industrial Microbiology & Biotechnology*.

[20] Sajedi RH, Naderi-Manesh H, Khajeh K (2005). A Ca-independent *α*-amylase that is active and stable at low pH from the *Bacillus* sp. KR-8104. *Enzyme and Microbial Technology*.

[21] Goyal N, Gupta JK, Soni SK (2005). A novel raw starch digesting thermostable *α*-amylase from *Bacillus* sp. I-3 and its use in the direct hydrolysis of raw potato starch. *Enzyme and Microbial Technology*.

[22] Ammar YB, Matsubara T, Ito K (2002). New action pattern of a maltose-forming *α*-amylase from *Streptomyces* sp. and its possible application in bakery. *Journal of Biochemistry and Molecular Biology*.

[23] Palmer T (2001). *Enzymes: Biochemistry, Biotechnology and Chemical Chemistry*.

[24] Zhang J-W, Zeng R-Y (2008). Purification and characterization of a cold-adapted *α*-amylase produced by *Nocardiopsis* sp. 7326 isolated from Prydz Bay, Antarctic. *Marine Biotechnology*.

[25] Uyar F, Baysal Z, Doğru M (2003). Purification and some characterization of an extracellular *α*-amylase from a thermotolerant *Bacillus subtilis*. *Annals of Microbiology*.

[26] Chakraborty S, Raut G, Khopade A, Mahadik K, Kokare C (2012). Study on calcium ion independent *α*-amylase from haloalkaliphilic marine *Streptomyces* strain A3. *Indian Journal of Biotechnology*.

[27] Gessesse A, Mamo G (1999). High-level xylanase production by an alkaliphilic *Bacillus* sp. by using solid-state fermentation. *Enzyme and Microbial Technology*.

[28] Arikan B (2008). Highly thermostable, thermophilic, alkaline, SDS and chelator resistant amylase from a thermophilic *Bacillus* sp. isolate A3-15. *Bioresource Technology*.

[29] Stamford TLM, Stamford NP, Coelho LCBB, Araújo JM (2001). Production and characterization of a thermostable *α*-amylase from *Nocardiopsis* sp. endophyte of yam bean. *Bioresource Technology*.

[30] Yang C-H, Liu W-H (2004). Purification and properties of a maltotriose-producing *α*-amylase from *Thermobifida fusca*. *Enzyme and Microbial Technology*.

[31] Mollania N, Khajeh K, Hosseinkhani S, Dabirmanesh B (2010). Purification and characterization of a thermostable phytate resistant *α*-amylase from *Geobacillus* sp. LH8. *International Journal of Biological Macromolecules*.

[32] Kikani BA, Singh SP (2011). Single step purification and characterization of a thermostable and calcium independent *α*-amylase from *Bacillus amyloliquifaciens* TSWK1-1 isolated from Tulsi Shyam hot spring reservoir, Gujarat (India). *International Journal of Biological Macromolecules*.

[33] Shafiei M, Ziaee A-A, Amoozegar MA (2010). Purification and biochemical characterization of a novel SDS and surfactant stable, raw starch digesting, and halophilic *α*-amylase from a moderately halophilic bacterium, *Nesterenkonia* sp. strain F. *Process Biochemistry*.

[34] Chakraborty S, Khopade A, Biao R (2011). Characterization and stability studies on surfactant, detergent and oxidant stable *α*-amylase from marine haloalkaliphilic *Saccharopolyspora* sp. A9. *Journal of Molecular Catalysis B: Enzymatic*.

[35] Roohi R, Kuddus M, Saima S (2013). Cold-active detergent-stable extracellular *α*-amylase from Bacillus cereus GA6: biochemical characteristics and its perspectives in laundry detergent formulation. *Journal of Biochemical Technology*.

